# RETRACTION: Long Noncoding RNA HCG11 Inhibited Growth and Invasion in Cervical Cancer by Sponging miR‐942‐5p and Targeting GFI1

**DOI:** 10.1002/cam4.70465

**Published:** 2024-12-11

## Abstract

**RETRACTION:** Y. Zhang, J. Zhang, L. Mao, X. Li, ‘Long Noncoding RNA HCG11 Inhibited Growth and Invasion in Cervical Cancer by Sponging miR‐942‐5p and Targeting GFI1’, *Cancer Medicine* 9, no. 19 (2020): 7062–7071, https://doi.org/10.1002/cam4.3203.

The above article, published online on 13 August 2020 in Wiley Online Library (wileyonlinelibrary.com), has been retracted by agreement between the journal Editor‐in‐Chief, Stephen Tait; and John Wiley and Sons Ltd. The retraction has been agreed upon following an investigation into concerns raised by a third party, which revealed an inappropriate duplication of image panels (Figure 2C) between this, and another article published previously by a different group of authors, in a different scientific context. The explanation and the partial raw data shared by the authors was deemed insufficient to address these concerns. The authors' institute has been informed but remained unresponsive. Thus, the editors have lost confidence in the presented data and consider the conclusions of this manuscript substantially compromised.

**RETRACTION:** Y. Zhang, J. Zhang, L. Mao, X. Li, ‘Long Noncoding RNA HCG11 Inhibited Growth and Invasion in Cervical Cancer by Sponging miR‐942‐5p and Targeting GFI1’, *Cancer Medicine* 9, no. 19 (2020): 7062–7071, https://doi.org/10.1002/cam4.3203.

The above article, published online on 13 August 2020 in Wiley Online Library (wileyonlinelibrary.com), has been retracted by agreement between the journal Editor‐in‐Chief, Stephen Tait; and John Wiley and Sons Ltd. The retraction has been agreed upon following an investigation into concerns raised by a third party, which revealed an inappropriate duplication of image panels (Figure 2C) between this, and another article published previously by a different group of authors, in a different scientific context. The explanation and the partial raw data shared by the authors was deemed insufficient to address these concerns. The authors' institute has been informed but remained unresponsive. Thus, the editors have lost confidence in the presented data and consider the conclusions of this manuscript substantially compromised.

